# Cyber-Physical-Social Awareness Platform for Comprehensive Situation Awareness

**DOI:** 10.3390/s23020822

**Published:** 2023-01-10

**Authors:** Irfan Baig Mirza, Dimitrios Georgakopoulos, Ali Yavari

**Affiliations:** School of Science, Computing and Engineering Technologies, Swinburne University of Technology, Melbourne, VIC 3122, Australia

**Keywords:** IoT, social media analytics, cyber-physical-social computing, semantic situation awareness

## Abstract

Cyber-physical-social computing system integrates the interactions between cyber, physical, and social spaces by fusing information from these spaces. The result of this fusion can be used to drive many applications in areas such as intelligent transportation, smart cities, and healthcare. Situation Awareness was initially used in military services to provide knowledge of what is happening in a combat zone but has been used in many other areas such as disaster mitigation. Various applications have been developed to provide situation awareness using either IoT sensors or social media information spaces and, more recently, using both IoT sensors and social media information spaces. The information from these spaces is heterogeneous and, at their intersection, is sparse. In this paper, we propose a highly scalable, novel Cyber-physical-social Awareness (CPSA) platform that provides situation awareness by using and intersecting information from both IoT sensors and social media. By combining and fusing information from both social media and IoT sensors, the CPSA platform provides more comprehensive and accurate situation awareness than any other existing solutions that rely only on data from social media and IoT sensors. The CPSA platform achieves that by semantically describing and integrating the information extracted from sensors and social media spaces and intersects this information for enriching situation awareness. The CPSA platform uses user-provided situation models to refine and intersect cyber, physical, and social information. The CPSA platform analyses social media and IoT data using pretrained machine learning models deployed in the cloud, and provides coordination between information sources and fault tolerance. The paper describes the implementation and evaluation of the CPSA platform. The evaluation of the CPSA platform is measured in terms of capabilities such as the ability to semantically describe and integrate heterogenous information, fault tolerance, and time constraints such as processing time and throughput when performing real-world experiments. The evaluation shows that the CPSA platform can reliably process and intersect with large volumes of IoT sensor and social media data to provide enhanced situation awareness.

## 1. Introduction

Advances in the cyber-physical-social computing systems, in terms of social sensing, networking, mobile, and cloud technologies, are blurring the boundaries between the physical, social, and cyber worlds and causing an explosion of big data that comes from diverse and heterogenous data sources such as physical, wearable, interconnected sensors from Internet of Things and social media platforms such as Twitter [1,2,3], Facebook [4], Reddit [5], Weibo [6,7], and TripAdvisor [8]. The information from these sources needs to be instantly turned into valuable knowledge and decision/action for enriching situation awareness. Situation awareness is a concept that was initially used in military services that involves knowing what is happening around us at any point in time. Endsley [9] defines situation awareness as the perception of elements in the environment within a volume of time and space, the comprehension of their meaning, and the projection of their status in the near future. While perception is being aware of the elements such as the sensor measurements and social media with respect to the decision maker’s goals. These elements when put together help the decision-maker form a holistic picture of the environment.

We define a situation as the collection of high-value information that contains values corresponding to different features that relate to ourselves, anything that we care for or are interested in. The key to understanding situations is the construction of information spaces that holds high-value information corresponding to a specific situation. An information space consists of data that is required to understand a situation; however, not all data characterises an information space. An information space is characterised by the high-value information corresponding to the specific situation and this high-value information needs to be identified, searched, or extracted from the huge volumes of data generated by IoT sensors and social media sources. The IoT sensor and social media information spaces constructed from the enormous amount of heterogenous data from IoT sensors and social media data sources, etc., can provide various perspectives of the same situation. Just like IoT, social media allows their users to post messages that share their observations on situations with others. IoT sensors are machines that monitor situations in the physical world and provide observations via the internet; whereas, social sensors are people monitoring and reporting their observation of situations via postings on social media.

On the other hand, the rapid emergence of cyber-physical-social systems as mainstream information systems [10], has opened new avenues for enriching situation awareness using large volumes of heterogenous data generated by IoT sensors and social media. Cyber-physical-social(CPS) computing system is defined in [11] as a “*system comprising cyber, physical, and social components, which exists or emerge through the interactions between those components. A CPS System comprises at least one physical component responsible for sensing and actuation, one cyber component for computations and one social component that allows smart devices to detect reason and objectify social interaction responses of humans”*. A CPSA platform is a particular type of system formed by all the interconnected devices and objects from IoT and social media that can collect and report information on a situation from the physical world in order to achieve situation awareness on the cloud. It should be noted that although the terms CPS [11,12,13] and IoT [14,15] have been used interchangeably in literature, there is a distinct difference between the two [13]. While CPS focuses on the interconnection between the physical, social, and cyber worlds, IoT focuses on the interconnection between sensors.

Today, IoT and social media data represent a significant (if not dominant) portion of the volume of data traffic in CPS systems and offer a great opportunity to increase the scope and accuracy of situation awareness using CPS systems. The volume of data generated by billions of interconnected IoT sensors and social media platforms has grown significantly over the last few years. The real-time availability of social media data makes it a valuable resource for understanding various situations. Its volume, velocity, unstructuredness, heterogeneity, and enormous volumes make it challenging to process such data [16]. The volume and velocity of social media posts tend to be extremely high during times of an event, making the filtering of relevant situational data a complex and challenging task. Filtering relevant situational data from social media data spaces is further complicated by the short, inconsistent nature of social media postings [8] and their high volumes make it time-consuming to filter relevant situational data. The volume of data generated by social media varies based on the extent of emergency events as the number of affected people and geographical area vary [17], and social media adoption (number of active users) in the affected area. This sometimes puts an additional processing load on the hardware components of the system and in case of disaster situations, increases the probability of failure [1]. Therefore, it is important that the systems that provide situation awareness reliably handle information.

Further, humans capture and report data that describes a situation in different formats in social media than what is captured and reported by IoT sensors for the same situation. Moreover, this becomes even more challenging when social media users use different vocabulary to report on the same aspects of a situation. Data in social media is different to the data from IoT sensors in semantics, syntax, and structure and very often the structure of social media data is unknown. It is important to combine data from disparate sources and translate the data into valuable information through the use of semantics [15,18,19]. To enrich situation awareness, Yuchen et al. [10] have identified that it is important to enable interaction between IoT sensors and social media information spaces in cyber-physical-social systems. Data in various formats present challenges in semantic integration [1,18] and also contains a high amount of noise, and hence, becomes even more challenging in times of disaster situations where the system resources are already constrained. Maguerra et al. [20] developed a situation-awareness system involving semantics and big data. However, their system considered social media data only.

In this paper we propose a novel cyber-physical-social awareness (CPSA) platform that distils high-value information from both IoT sensor and social media information spaces by semantically describing and integrating the information extracted from sensor and social media spaces using a semantic situation model for enriching situation awareness. Unlike traditional situation awareness systems, the CPSA platform considers information from both IoT sensors and social sensors and then combines these information spaces [2,6,7,16,17,21,22,23,24,25,26] based on semantic situation models in a cloud environment for enriching situation awareness. However, such a CPSA platform is far from realised due to challenges in semantically integrating information, i.e., semantic integration and fault tolerance when harvesting and analysing data. This is due to the characteristics of the CPSA platform that require harvesting heterogeneous information, homogenising the features using a semantic situation model and provides an efficient intersection of the features for improved situation awareness. To illustrate and assess situation awareness improvements using the CPSA platform, we designed and implemented a novel CPSA platform for intersecting sensor and social information spaces using a situation model. The CPSA platform is highly scalable, utilises distributed cloud computing services, for semantically describing the situation and is fault tolerant when reliably handling large volumes of social media and sensor data for enriching situation awareness. This paper includes the following novel contributions:

- A Semantic Framework for describing situations of interest.
- A CPSA platform that uses the situation models to integrate and intersect information from sensors and social media.
- A proof-of-concept implementation of CPSA platform including a cloud-based visualisation dashboard for monitoring the health and performance of the CPSA platform in real time.
- An experimental evaluation that shows the benefits of the CPSA platform for enriching situation awareness.
- A fault tolerance-related evaluation of the CPSA platform that shows the CPSA platform can reliably handle large volumes of social media and sensor data.

The rest of this paper is organised as follows: Section 2 presents the related work and various state-of-the-art systems that provide situation awareness in the IoT sensor information space, systems that provide situation awareness in social media information space, and related work in systems that provide situation awareness in combined information space. Section 3 presents the benefits of the proposed CPSA platform in (a) semantically describing and integrating information from sensors and social media information spaces and (b) intersecting the information for enriching situation awareness using a sample scenario of bushfire situation awareness. Section 4 describes the architecture of the proposed CPSA platform. Section 5 discusses harvesting heterogeneous sensor and social media data in the CPSA platform using cloud based big data tools. Section 6 discusses feature extraction and mapping to a semantic situation model in the CPSA platform. Section 7 discusses information fusion in the CPSA platform to enrich situation awareness. Section 8 discusses the implementation methods and results from the evaluation of the CPSA platform when performing a situation model-based intersection of sensors and social media information spaces. Section 9 provides a discussion of the CPSA platform and Section 10 concludes the paper and describes the potential future research directions.

## 2. Related Work in Situation Awareness Systems and Cyber-Physical-Social Computing

In this Section, we present related work in situation awareness systems and Cyber-physical-social Computing. Related research for providing situation awareness considers either information harvested by IoT sensors (e.g., sensors onboard mobile phones, vehicles [27], camera sensors, UAV’s [28], IR cameras, and inertial sensors) or social media (e.g., posting in Twitter [1,2,3], Facebook [4], Reddit [5], Weibo [6,7], and TripAdvisor [8]). Situation awareness systems using combined information spaces (both sensor and social media) have also been recently proposed for identifying the utility of IoT sensor features [6], augmenting the low temporal resolution of satellite imagery [2], and providing situation awareness of other situations such as floods. Table 1 summarises the existing literature in terms of the situation awareness applications, their data sources, and the use of semantics when using either sensor or social media spaces and both sensor and social media spaces for providing situation awareness.

When providing situation awareness, it is important that the situation-awareness systems are able to ensure high data availability even during blackouts or unplanned or unexpected infrastructure upgrades or maintenance or impairment. Pasandideh et al. [12] have identified that the mapping and fusion of heterogeneous information between IoT sensors and social media information spaces create challenges in terms of modelling and designing a CPSA platform. Fault tolerance is identified as the capability of a system that enables the system to continue its operations in the event of a fault [36,37]. Fault tolerance is regarded as a critical component [1,38] of big data systems involving sensors and social media for provisioning high data availability and system performance. The demand for achieving efficient fault-tolerant systems, i.e., the systems that can reliably handle large data volumes, to ensure high data availability has increased rapidly [38] with the significant adoption of IoT sensors and social media platforms for providing situation awareness [1,6,17,21,22,23]. Achieving a good level of tolerance is challenging [38] due to the demand of big data systems in terms of their performance and resource consumption. Various solutions have been proposed in literature to reliably handle information [36,37,38,39], however, they involve either sensor or social media data. Shah et al. [1], have identified the need for systems to be equipped with capabilities such as consistent backups and cloud-based mechanisms that allow support for distributed computing.

Cyber-physical-social Systems commonly integrate various resources from physical, cyber, and social worlds while also providing an efficient interaction of these resources [40]. In existing CPS systems, the IoT sensor and social media information spaces are connected to their respective cyber systems which then forms the idea of s platform-based approach for system-level design [12]. Reine et al. [13] have identified the need for systems in healthcare applications as source information from wearable sensors and health records. For instance, in the case of patients with critical diseases and elderly people that need to be consistently monitored, it would be very beneficial to include information from wearable sensors that collect the patient’s physiological data, such as temperature, blood pressure, etc., along with the diagnosis information from doctors and nurses.

In literature, systems based on either social media and/or sensors have been built to provide situation awareness. For instance, ConTaaS was introduced in [34], as a contextualisation architecture for contextualising Internet-scale IoT data and facilitating the development of efficient situation-awareness applications using IoT sensor data. Shah et al. [1] proposed a reference big data architecture consisting of Hadoop and Spark for situation awareness in smart city environments. Twitris [33] utilised data from Twitter to semantically enrich and classify situations. Imran et al. [41] developed AIDR, the Artificial Intelligence for Disaster Response system that uses Twitter data for situation awareness. Tweedr [42]’s system provided situation awareness using Twitter data. Computer vision models have been widely used for feature extraction from IoT sensors to provide situation awareness. Deep learning algorithms based on convolution neural networks have been used in situation-awareness systems to detect people, i.e., segregate people from the background with high accuracy in real-time, estimate their physical distance and count the social distancing violations. While existing situation awareness systems are aimed at providing situation awareness solutions using either IoT sensor or social media information spaces [1,43,44,45,46] and the situation awareness systems that include both sensor and social media data sources are referred to as combined information spaces [16,21,23], none of the existing systems supports the capability for semantically describing situations using semantic situation models when enriching situation awareness for reasons discussed in Section 3. Researchers [1,11,12,13,20,47] have identified semantic integration and reliable handling of sensor and social media information, i.e., reliable handling as one of the key challenges when designing systems for situation awareness. In this paper, we propose a novel CPSA platform to address the challenges in semantic integration, and reliable handling of data large volumes when harvesting the data from IoT sensors and social media information space using semantic situation models for providing situation awareness.

## 3. Benefits from Intersecting and Fusing Sensor and Social Media Information Spaces

In this Section, we discuss the benefits of intersecting and fusing sensor and social media information spaces using a CPSA platform for semantically describing situations using situation models, and intersecting information for enriching situation awareness in Section 3.1, as well as the challenges in semantic integration in Section 3.2.

### 3.1. Motivating Example—Identifying Potential Bushfire Hotspots and Improving Bushfire Emergency Management

Bushfires are complex processes that find a natural occurrence in Australia [48]. Bushfires can spread very quickly, become devastating upon contact with homes, infrastructure and people and result in significant loss of human and animal life. Fire danger ratings are commonly used to indicate the possible consequences of a fire situation. In the state of Victoria, fire danger ratings are updated twice daily at 5.30 a.m. and 4 p.m. and are calculated based on predicted conditions such as temperature humidity, wind, and dryness of the landscape. However, fuel loads such as fallen tree limbs, small fallen branches, twigs, leaf shred, shrubs, etc., elevate the bushfire at a rapid pace. The bushfires tend to become more intense as the density of the fuel load increases. It is very unlikely to prevent a bushfire before it occurs naturally. However, our response to bushfires can improve by having a better understanding of the surroundings and situation as it progresses. This could help in improving our decision-making in terms of targeting specific vulnerable areas, mobilising teams to fight bushfires and helping reduce the bushfire’s impact on human and animal life and infrastructure. Significant changes in weather conditions have been found to have contributed to a fire consequence and meteorological information has been identified as the key input to fire prediction. IoT-based weather sensors, which could provide information on humidity, air temperature, images and videos from sensors onboard satellites, drone cameras, social media postings on Twitter, etc., can be used to provide first-hand information about the bushfire situation. For situation awareness of bushfire situations, the weather conditions in the state of Victoria are constantly monitored by the Bureau of meteorology using infrastructure such as IoT-based weather sensors at various locations across the state. Further, the information from drone cameras, street cameras, and remote sensing satellites could also be utilised to gather supplementary information, as well as from social media postings where human sensors report their observations.

**Figure 1:**
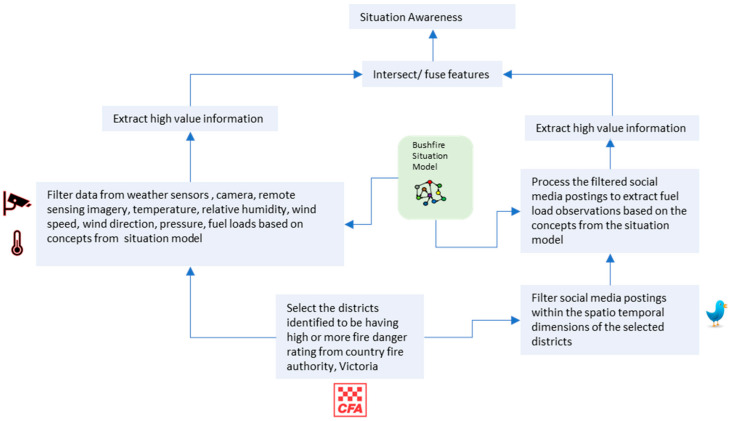
Illustration of the physical infrastructure and data analysis tasks for identifying potential fire hotspots and improving bushfire emergency management in a CPSA platform.

Although the information from IoT sensors, drones, street cameras, and remote sensing satellites can be used to determine bushfire hotspots, there could be situations when there is limited sensor coverage, sensor malfunction, etc., in the bushfire area. In this case, the first-hand information being provided by the sensors is no longer available to manage bushfire rescue efforts. However, with social media being widely used by human sensors to report their observations, the information from social media could be used to compensate for the information sparseness from IoT sensors. However, with social media postings being semantically and syntactically different they are also unstructured. Situation models can be used to provide a description of the situation beforehand and these situation models could help in extracting and homogenising information from social media postings. The homogenous information then needs to be semantically integrated with the information from IoT sensors and then intersected for enriching situation awareness. Such an intersection allows for richer information to be provided. Figure 1 illustrates the physical infrastructure and data analysis tasks that need to be performed in the CPSA platform for enriching situation awareness by identifying potential fire hotspots and improving bushfire emergency management.

A CPSA platform could be considered for enriching situation awareness of bushfire situations. The CPSA platform should utilise the data from the IoT sensors, social media, and authoritative government sources such as the country fire authority and perform the following tasks to get first-hand information on bushfire hotspots, injuries to human and animal life, and infrastructural damage: (1) utilise a situation model for providing semantic descriptions of bushfire situation. (2) Select the districts reported by the country fire authority as having a high fire rating in the state of Victoria. (3) Perform an initial analysis of atmospheric conditions by analysing data from IoT sensors that report on wind direction and speed, humidity, air temperature, etc. (4) Utilise the supplementary data sources in drone cameras to extract supplementary information on fuel components. (5) Then, extract and provide high-value information via semantic integration of information based on the descriptions provided by the situation model. (6) Filter relevant social media postings within the spatial–temporal dimensions of the IoT sensor in the districts identified as having a high fire rating. (7) Extract high-value information related to fuel load components based on the situation model descriptions and then perform a semantic integration. (8) Intersect the high-value information extracted from IoT sensors and social media postings to enrich bushfire understanding. Here, the information from social media can be used to supplement the information from IoT sensors by providing more information in terms of human and animal life and infrastructural damage, such as roads, property, vehicles, and people reported being trapped. Semantic integration allows the heterogenous data from IoT sensors and social media posting to have a unified view of the bushfire situation.

### 3.2. Semantic Integration

Simple situations involve only space, time, or a single basic concept or basic data type (e.g., a keyword in social media or a value of a basic data type such as pressure) [35]. Complex situations expand beyond common spatial–temporal definitions and in order to be understandable, they have to be modelled [35]. There are many situations such as rainfall, natural disasters, and bushfires where it is not possible to utilise data from IoT [28,49] due to the reasons discussed in Section 3.1. Researchers in [1,6,7,16,22,23,35], recognised the need for utilising social media data to supplement data from IoT sensors for enriching situation awareness. However, the data from IoT sensors and social media information spaces is heterogenous and can present itself in structured and unstructured ways. Examples of structured data include geographical location coordinates, sensor data from temperature, wind, humidity sensors, etc., and unstructured data include social media postings, video from data from sensors, etc. It is important that this data is semantically integrated for enriching situation awareness. In [23], the integration between sensors and social media information spaces was achieved by converting Twitter messages into rainfall values based on the frequency of geolocated tweets containing flood-related keywords obtained for cumulative periods (20, 30, 40 min). Such transformations, after identifying relevant social media posts, commonly consider the frequency of search keywords appearing in these social media posts to construct a social media information space feature which is similar in datatype to the sensor information space feature. Feature translation based on the above techniques, i.e., the frequency of social media postings is driven by the adoption of social media in the area of interest. Further, social media activity is also driven by the spatial distribution of social media users which could potentially introduce some when analysing the situation of interest.

Another challenge in semantic integration involving sensor and social media information spaces arises when extracting high-value information, especially from social media information spaces. This is challenging due to the different vocabulary used by people to report on the same situation. Further, false information and unrelated information may still exist, which may affect the quality of the information being extracted. Aside from this, social media activity is generally associated with the geographical and demographical distribution of users; the social and spatial heterogeneity in social media postings might also bring some unwanted information during the extraction of useful information and analysis of situations. In order to overcome these challenges imposed by the heterogenous and noisy nature of social media information space, a solution for supporting situation awareness enrichments using both sensor and social media information spaces should also be capable of extracting useful information to ensure the extraction of high-value information relevant to situations. A situation model provides a formal description of the situation, using an ontology composed of classes, subclasses, objects, data properties, etc. [35]. A situation model can efficiently describe situations using sensors and social media information spaces. Ontology-based situation models can utilise the classes, subclasses, and relationships to model situations of interest using both sensors and social media information spaces, and also help in overcoming challenges such as changes in diction, word structure, and user expectations in social media information spaces. For example, as discussed in the motivating example of bushfire emergency management in Section 3.1, Bushfire situations might change very quickly in the presence of fuel loads such as fallen bark, leaf litter, and small branches accumulating in the landscape, twigs, leaf litter, etc., as the greater the fuel load, the hotter and more intense the fire. Ontology-based techniques are flexible enough to model the potential causes of bushfires as concepts in the situation model and these concepts can be identified from the sensor and social media information spaces. Therefore, a solution for enriching situation awareness using the proposed CPSA platform which fuses the sensor and social media information spaces based on a situation model needs to be able to semantically integrate information between sensors and social media information spaces.

## 4. Architecture of Cyber-Physical-Social Awareness (CPSA) Platform

In this Section, we propose a reference architecture for a novel CPSA platform that aims at enriching situation awareness by semantically describing situations and intersecting high-value information from the IoT sensor and social media information spaces using a situation model. As we discussed in Section 2, several CPS-based systems have been proposed for improved situation awareness combined with information spaces [1,47]. However, to the best of our knowledge, no CPS system has focused on semantic integration of sensor and social media information spaces for enriching situation awareness and reliable handling of large volumes of social media and sensor data. When designing the architecture of the proposed CPSA platform, we have included the following considerations during the design process.

1. The architecture of the CPSA platform should have the capability to connect to any potential data source in IoT sensors and social media platforms, from the cloud environment.
2. The CPSA platform architecture should support the semantic description of situations and the intersection of high-value information from IoT sensor and social media information spaces.
3. The CPSA platform should be able to provide data harvesting capabilities in a fault-tolerant manner when harvesting data from sensors and social media sources at the same time.
4. The physical infrastructure of the CPSA platform should be scalable to handle different data processing and analytical workloads.
5. The CPSA platform should be able to export the situation awareness results or cloud infrastructure health information via cloud APIs for end users.

**Figure 2:**
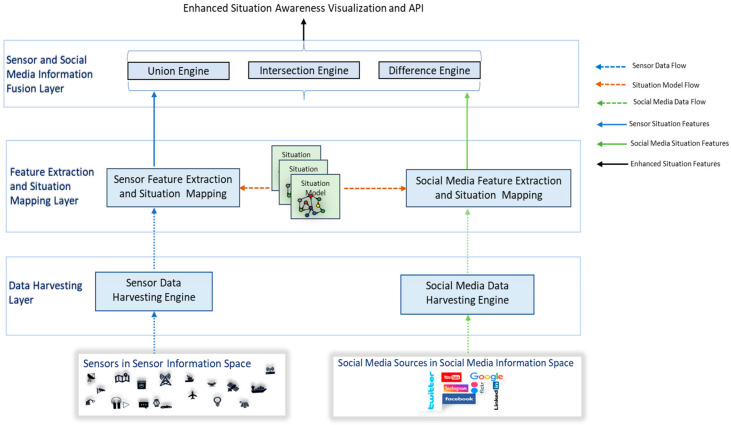
Architecture of CPSA platform for improving situation awareness using sensor and social media information spaces based on a situation model.

As shown in Figure 2, the proposed CPSA platform is split into three layers between the data sources and the API endpoints, i.e., (1) Data-Harvesting Layer, (2) Feature Extraction and Mapping Layer, and (3) Sensor and Social Media Mapping Layer. Section 5, Section 6, Section 7 and Section 8 describe the functions of each of these layers in detail.

## 5. CPSA Data Harvesting

In this Section, we discuss data harvesting using the CPSA platform. IoT sensors and human sensors (also known as social sensors [50]) are common sources of information in cyber-physical-social computing systems [11,13]. IoT sensors are physical devices that monitor situations in the physical world and provide observations via the internet; whereas, human sensors are the people monitoring and reporting their observation of situations in the social world via postings on social media. The data-harvesting layer in the CPSA platform can support harvesting data from a wide variety of IoT and human sensors such as camera, IR, temperature, pedestrian sensors, and Twitter, Facebook, Weibo, Reddit, etc., respectively, using the sensor data-harvesting and social media data-harvesting engines. Within the CPSA platform, both these engines reside within the data-harvesting layer and use a variety of Kafka Producers as discussed in Section 8 to harvest data from IoT and social media data sources. The Kafka producers in the data-harvesting layer are responsible for harvesting the data from various IoT and social media data sources. In the case of harvesting data from social media platforms, the Kafka producer applications in the social media harvesting engine harvest data by connecting to social media sources such as Twitter using the Twitter streaming API and support a wide variety of keywords, hashtags, geotags, or a combination of these to filter social media postings and then publish the tweets into Kafka topics which are later consumed by the feature extraction and mapping layer. Keyword-based filtering, which is the most common method for filtering situational data consists of a set of collection of terms that are typically assumed to describe efficiently a situation. Hashtag—a word or an unspaced phrase preceded by a # symbol is also frequently used to describe and track a situation, a topic, or an aspect of the event to help posts stand out in social media. Geotag filters consists of a set of location parameters such as location coordinates or place names for filtering data from social media data spaces. When using geotag filters, the queries for identification and extraction include matching the data from social media data sources to the location parameters for retrieving social media postings that are geotagged to the location parameters. At this stage, the harvested data can either be structured or unstructured and also in various formats such as CSV, XML, JSON, etc., depending on the data source.

## 6. CPSA Feature Extraction and Mapping

In Section 6, we discuss the feature extraction and mapping using the CPSA platform. Section 6.1 presents a discussion on the situation modelling; Section 6.2 discusses sensor feature extraction and mapping engine; and Section 6.3 is on feature extraction and mapping using social media. The primary aim of the feature extraction and mapping layer is to process the raw data from the data-harvesting layer and extract relevant features based on the situation model descriptions. Although feature extraction is independent of the techniques used for situation awareness, good features provide decreased training times and reduce overfitting in such algorithms whereas a redundant and high number of features negatively impact model performance and also increases the complexity of situation awareness tasks. For example, the smart parking recommender application which implemented the ConTaaS [34,51] architecture, applied contextual filter operation by considering data from machine sensors only. In this, the data received from a parking sensor located in a particular location (e.g., a parking space in a Melbourne suburb) was excluded from further data processing and related queries whenever there is no particular user looking for parking in that particular location. The inclusion of features in an information space depends on the situation that is being understood, and hence, having a combined information space with an optimal set of features has a significant impact on improving situation awareness. In the smart parking recommender application, the features from social media information spaces can be leveraged to provide recommendations on parking availability, the time taken to reach the parking spot or to exit, or pricing information of nearby parking spots in case of parking spot unavailability in the current location. Including this information can greatly enhance the user’s search. The concepts such as pricing information or travel time extracted from social media can be used to further contextualise the high-value information. In the CPSA platform, the feature extraction and mapping layer consist of three engines, a situation modelling engine, a sensor feature extraction and mapping engine, and a social media feature extraction and mapping engine.

### 6.1. Situation Modelling

The Situation Modelling Engine is used to create and store situation models that describe specific situations. Mirza et al. [35] define a situation *as “the collection of all the features, their relationships and situation model as an ontology or a fragment of an ontology that possibly includes classes, subclasses, properties, etc., that are necessary to provide a formal description of the features of a situation of interest”.* Gruber [48] defines ontologies as a “formal explicit specification of a shared conceptualization”. By formal explicit specification, it is expected that the ontology is understandable and readable by machines. Shared implies the community consensus towards the ontology and conceptualisation refers to the concepts and properties used to represent knowledge in a specific situation. An important characteristic of the situation model lies in its ability to describe a situation from heterogenous sensors and capture their relationships [35]. While ontologies commonly deliver semantic integration [40] of heterogeneous and unstructured data from IoT sensors and social media information spaces, they can also be used to design and develop situation models that are compact and require less effort and time to be developed and thereby provide means to create sustainable ontology-based situation models that can be used for enriching situation awareness.

### 6.2. Sensor Feature Extraction and Mapping Engine

The goal of the sensor feature extraction and mapping engine is to read the raw sensor data harvested by the data-harvesting layer, clean and transform the data, extract meaningful features based on the situation model descriptions, and map the sensor data to the concepts in the situation model. A feature is a specific, observable, and measurable property (characteristic) such as height, colour, etc., of something that we are interested in. For example, consider a camera sensor, which monitors people’s movements, and the goal is to create a feature that measures the average distance between two people. The sensor feature extraction and mapping engine needs to process the video data stream, and produce relevant features based on the situation model descriptions. The first step in this process is to use existing best-in-class computer vision algorithms [52,53] to identify objects in a video stream and then filter the objects that are people. The next step is to compute the Euclidean distance between the identified people and add the distance measurements as values to the feature. The relevant features provided by the machine sensor feature extraction engine, in this case, would include the location, time, and the average distance between people.

### 6.3. Social Media Feature Extraction and Mapping Engine

The goal of the social media feature extraction and mapping engine is to read the social media postings harvested by the data-harvesting layer and extract meaningful features based on the situation model descriptions and map the social media data to the concepts in the situation model. For example, discuss the various tasks performed by the social media feature extraction engine for extracting relevant features from raw social media postings include, noise removal, normalisation, tokenization, named entity recognition, parts of speech tagging, etc. In the social media feature extraction and mapping engine, the first step in feature extraction is processing social media postings to remove noise. Noise removal includes removing stop words, special characters, punctuations, acronyms [54], emoticons [54], HTML formatting, etc. Stop words are those words that do not add any semantics to the sentence and their removal does not affect the feature extraction. Normalising the abbreviations, mis-spelled, and of-vocabulary words into their standard form also helps in removing noise. For example, 2 mrw to tomorrow, b4 to before, otw to on the way, etc. The raw postings after noise removal are converted into a set of tokens. Tokenization involves breaking up raw postings into atomic units called tokens. These atomic units often embed contextual information and are considered a semantic unit for data processing. Various tokenization techniques are out of the scope of the discussion of this paper and the reader may refer to a study on various tokenization techniques in [55]. For instance, if the situation model requires that the concepts extracted from the posting be associated with a time and location, then the timestamp associated with a social media posting and the corresponding geotag can be used for this purpose and similarly, with named entities such as people, place names, natural phenomenon, and parts of speech. NER and POS tagging can be applied to extract the entities and concepts. Entities are real-world objects such as person, location, and organisation names. Named entity recognition (NER) is the task of detecting and classifying named entities (NEs) within texts into predefined classes, such as person. Parts of speech (POS) Tagging is the process of assigning relevant parts of speech (noun, pronoun, verb, adverb, or adjective) to each word in a sentence [56]. POS tagging could also help distinguish features such as noun: smoking (instead of a verb: smoking). The word “smoking” is being used in a social media post as a noun (“Smoking can lead to cancer”) instead of a verb (“The singer put on a smoking performance”). The relevant features extracted by the social media feature extraction and mapping engine, in this case, would be the location, time, entities, and concepts.

## 7. CPSA Sensor and Social Media Information Fusion

In this Section, we discuss the information fusion in sensor and social media using the CPSA platform. The sensor and social media information fusion layer in the CPSA platform is aimed at providing fusing high-value information from the sensor and social media information spaces. As we discussed in Section 6, Ontologies can be used to construct semantic situation models to describe situations, for identifying and extracting relevant features from the sensor and social media. Features from sensor information space are well structured and easily identifiable when compared to social media. From social media, relevant features can be identified and extracted from social media postings by combining various techniques such as parts of speech tagging [2,56,57,58,59], named entity recognition [56,60,61], and linguistic features [5] such as character n-gram, word n-grams. In addition to this, extracting geolocation information from social media postings could also help in identifying various spatial features at different levels of a space hierarchy such as names of countries, states, and cities, as well as street addresses and intersections, or even narrowed further to highways and specific places such as parks and schools. This could lead to a possibility of an overlap of similar features between sensors and social media information spaces which could provide complimentary, or in some cases contradictory, information. The Union, Difference, and Fusion Engines are aimed at handling such kinds of information.

## 8. Implementation and Evaluation of CPSA Platform

In this Section, we discuss the implementation and evaluation of the CPSA platform. More specifically, in Section 8.1, we discuss the implementation of the data-harvesting layer, the health management of the data-harvesting layer in Section 8.2, the feature extraction in Section 8.3, and the fusion layer in Section 8.4. Section 8.5 presents the evaluation of the CPSA platform in terms of semantic integration in Section 8.5.1 and the reliable handling of large volumes of sensor and social media information in Section 8.5.2.

### 8.1. Implementation of the Data-Harvesting Layer in the CPSA Platform

**Figure 3:**
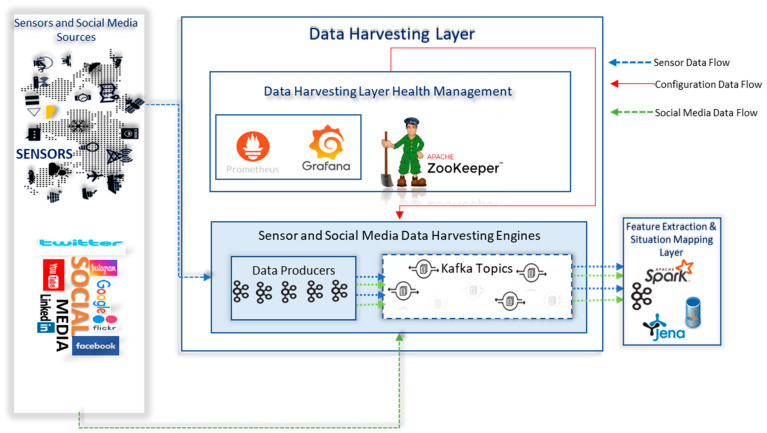
Implementation model of data-harvesting layer of CPSA platform.

As shown in Figure 3, the data-harvesting layer consists of Sensor and Social Media Data-Harvesting Engines which are responsible for harvesting the data from IoT sensors and social media data sources. These engines use Apache Kafka clusters for harvesting data. Apache Kafka is a fault-tolerant, highly scalable, and available open-source distributed streaming platform that can be used to store and process data streams. It primarily consists of topics, producers, and consumers. Topics are logical entities where data records are published by producers and consumers read data records from topics. In Kafka, messages are written to topics which are distributed across partitions. This process is commonly referred to as writes. Each message is assigned an offset id in the partition which is typically incremented and can never go back to zero. The writes process is immutable as once data is written to a partition, it cannot be changed. For example, if we write offset 7 in partition 3, it can never be updated or swapped, and a new message cannot be written on the same offset. It should be noted here that message ordering is guaranteed only within a specific partition and not across partitions and data. In the data-harvesting layer, we have three brokers, Data-Harvesting Engine 1, Data-Harvesting Engine 2, and Data-Harvesting Engine 3. It should be noted that the partition number and broker number do not have any relationship and Kafka randomly assigns partitions to brokers with topics being spread across brokers which are again distributed by Kafka.

We created various topics to store weather-related observations from automatic weather stations, incidents from the country fire authority and tweets from Twitter with a partition count of 8, *replication factor* of 3, min *insyncreplicas* as 2, and *unclean.leader.election.enable* = false as the topic settings. For example, we created a topic “*bom_weather_topic_hash*” to store the weather-related observations from automatic weather stations maintained by the bureau of meteorology. This topic has eight partitions spread across three brokers and it is possible that one broker does not hold a similar volume of data in the partitions as on other brokers and may have more than one partition of the same topic. We ensure fault tolerance by utilising the topic replication factor which defines how many times a topic should be replicated. We set the replication factor to three to maintain two copies on two different brokers. With this replication factor, the producer and consumers can still tolerate two brokers going down at the same time. For example, using this design we can afford to perform maintenance on one broker and in the event of another broker being lost during maintenance, we can still continue to serve data. In the event of a broker being down, the other brokers can continue to serve data and the replication ensures that the data is not lost. To enable replication, every partition needs to have a leader and *insynreplicas* (ISR’s) and only one broker can be a leader at a time for a particular partition. In this example, for partition 0, the partition leader is broker Kafka3 and is typically responsible for receiving and serving the data. The other brokers, Kafka1 and Kafka2, synchronise data with the Kafka3. The zookeeper manages the leaders and the ISR’s for each partition. The leader election process is initiated whenever a broker is lost, and leadership is transferred back to the broker once it is back.

### 8.2. Health Management Implementation Model of Data-Harvesting Layer in the CPSA Platform

To manage the health of sensor and social media data-harvesting engines, we designed and implemented the data-harvesting layer health management component. This component makes use of Apache zookeeper for configuration management, and Prometheus and Grafana dashboards for managing the health metrics and visualising the health of the data-harvesting cluster. We designed a three-node zookeeper cluster with one leader and two followers for configuration management of the brokers in the data ingestion layer. Zookeeper is distributed key-value store with a voting mechanism and provides multiple features to support distributed applications. It is widely used by various distributed big data systems [62] such as Hadoop, Kafka, etc., for managing their configuration. A zookeeper quorum is an ensemble of machines for the configuration management of distributed systems. To ensure low latency in zookeeper performance, we removed the ram swap and made sure all zookeeper instances are in the same region and isolated zookeeper instances from other processes. We set the zookeeper properties of *Maxclientcnxns* as 0 to support an unlimited max number of connections, *tickTime* as 2000 which indicates heartbeats for every 2 s, *initLimit* as 10 for initial synchronisation and a *syncLimit* of 5, *initLimit* of 10, and *tickTime* of 2 s indicate that a time of 20 s can be used for the initial synchronisation in the case of a fail and *SyncLimit* of 5 allows us to pass five syncs between a request and acknowledgement in case of timeout which means 10 s after five ticks we don’t get a sync then zookeeper will fail. These settings define a timeout and latency and the current combination along with a reliable network from nectar research cloud, Australia, which so far hasn’t allowed the zookeeper quorum to fail. The zookeeper design allows brokers to be connected to different zookeeper servers while the leader responsible for writers and followers for reads. Zookeeper provides Kafka notifications whenever events such as losing a broker or creating or deleting topics.

### 8.3. Implementation Model of Feature Extraction and Situation-Mapping Layer in CPSA Platform

Traditional data-processing systems consider single instances (CPU’s) for feature extraction, machine learning workloads using Apache Spark [63] that can be used to achieve situation awareness. However, with the advent of big data multiple cores, Graphics Processing Units (GPUs) are increasingly utilised for performance enhancement. Although GPUs are significantly faster [64], they are cost-intensive and are not economically feasible. Therefore, it is important that the single instances or instances on cloud referred to as EC2 instances are optimised and leveraged for distributed processing to handle high data volume. Apache Spark is a big data analytics engine for the large-scale processing of graph data, data that requires incremental computation and streaming data [65]. Spark supports various dynamic workloads for distributed applications written in languages such as Scala, Python R, and Java. The Spark engine consisting of SparkSQL, structured streaming, machine learning, and graph-processing libraries can be provisioned to run by itself or using various cluster deployment modes such as standalone, Apache Mesos (currently deprecated) [65], YARN, and Kubernetes. The standalone is packaged with Spark core and fulfils the requirements of a dedicated cluster. It can be managed with Apache zookeeper to ensure fault tolerance. Mesos is a general-purpose cluster manager for managing and distributing resources such as memory, CPU, network bandwidth, etc., between multiple applications on the cluster and facilitating multi-tenant and heterogenous workloads. YARN (Yet Another Resource Navigator) is a monolithic scheduler that can be used for managing cluster resources and job scheduling is well suited to stateless batch jobs with long run times and integrates well with existing Hadoop clusters. However, for stateful services such as database queries, YARN is not well suited. The standalone deployment mode is encapsulated within the Spark Framework and is commonly implemented to deploy Spark on a private cluster. Spark provides a SparkContext as a way of connecting to the cluster and allocates resources across applications. From Spark 2.0 onwards SparkSession replaced SparkContext and serves as a connection point for Spark Applications. After establishing a connection, applications typically run as independent sets of processes and their execution is coordinated via a SparkContext object known as the driver program [65]. The driver program listens and accepts connections and also analyses, distributes, and schedules work tasks across the executors and maintains information on the Spark applications. The executors are responsible for executing the work that the driver program has assigned, in the form of tasks. In essence, each executor is responsible for receiving and executing the code assigned to it by the driver of the tasks and reporting the state of task computation back to the driver program. It is important to ensure that the config object in spark.driver.port is accessible from the worker nodes in the cluster. Executors are an independent set of processes that run on the worker nodes and are responsible for computations on the cluster nodes and storing application-specific data. Worker nodes typically launch more than once executor processes in separate JVM’s based on the memory and cores. Spark acquires and releases executors as and when they are needed by the applications. Applications are assigned their exclusive set of executors for the duration of their execution and the executors commonly receive tasks from SparkContext to run in multiple threads in different JVMs’. However, the data within an application cannot be shared across multiple applications unless it is written in a storage layer.

As per the recommendations of Apache Spark [65] for memory allocation, we have allocated 75% of the memory as the maximum limit, with the actual memory needed being application dependent. The other recommended settings in terms of the minimum number of cores per machine are required to be 8–16 cores/machine. In addition to the cores, the memory settings we currently set as the parameters *SPARK_DRIVER_MEMORY* = 32 G and *SPARK_EXECUTOR_MEMORY* = 10 G. The Spark monitoring UI of the driver nodes accessible through port 4040 can be used to monitor the memory usage. The same UI can also be used to monitor the amount of data being moved in the Spark ecosystem. Spark uses cluster managers for scheduling applications, and a fair scheduler for scheduling resources within individual SparkContexts’. As the cluster we deployed runs in standalone mode, the cluster managers follow a static partitioning of resources, where each application is allocated the maximum amount of resources in a FIFO sequence and attempts to make use of all the available nodes. Spark provides many ways in which performance improvements be achieved such as by (a) controlling cores and (b) controlling memory use. For instance, the *spark.cores.max* configuration property can be used to limit the number of nodes used by a given application, or the setting *spark.deploy.defaultCores* can also be modified. The memory can be controlled via the application’s *spark.executor.memory* setting.

### 8.4. Implementation Model of Sensor and Social Media Information Fusion Layer in CPSA Platform

The sensor and social media information fusion layer consists of an EC2 instance built on top of Apache Jena framework. Apache Jena is an open-source framework for building semantic web and Linked Data applications that can be used to provide situation awareness. The framework is composed of different APIs interacting together to process data from the sensor and social media feature extraction and mapping layer. The RDF API allows for the creation of RDF graphs which represented the annotated sensor and social media data based on the situation model descriptions. This data is then serialised in the form of triples and can support common formats such as RDF/XML or Turtle, etc. The ontology API in the Jena framework provides the support to handle ontologies and the Inference API allows reasoning over the triples using built-in OWL and RDF reasoners [66]. Apache Jena Fuseki provides the SPARQL server capabilities and works as a Java web application. We implemented Fuseki as a single-system webapp with a combined user interface to both administration and querying purposes.

### 8.5. Evaluation of the CPSA Platform

This Section describes the system capabilities in terms of semantic integration and reliable handling of large volumes of IoT sensor and social media data in the CPSA platform by focusing on weather-related situation understandings as a use case. We describe the operational steps that we followed for extracting IoT sensor data from various weather stations across the state of Victoria and their related social media postings. Then, we discuss the semantic integration process that translates the IoT sensor data and social media data into high-value information and annotates the high-value information based on the concepts in the IoT sensor (SOSA) ontology and converts it into triples as well as storing the triples to the Sensor and Social Media Information Fusion layer which contains a cloud-based Apache Jena Fuseki triple store. We conclude by demonstrating the capability of the CPSA platform in reliable handling of large volumes of IoT sensor and social media data.

#### 8.5.1. Semantic Integration in CPSA Platform

The huge volumes of data harvested from various sensors and social media postings are in a variety of formats. To handle semantic integration in the CPSA platform, we implemented the situation model for weather-related situations developed in [35]. We then applied the semantic similarity matching techniques developed in [35] and mapped the observable property to a social media posting. For example, specific keywords extracted from social media postings that are synonymous with sosa: ObservableProperty such as pressure. This technique makes use of token similarity to estimate the similarity of a social media posting to sosa: observable property. A detailed discussion of the feature extraction and mapping techniques is out of the scope of this paper. The high-value information from social media information space is then transformed as triples and stored in an Apache Jena Fuseki triple store within the Sensor and Social Media Information Fusion Layer in the CPSA platform.

The Sensor Feature Extraction and Mapping Engine and Social Media Feature Extraction and Mapping Engine in the Feature Extraction and Situation-Mapping layer perform the transformation process required to ensure semantic integration. The process includes reading the data, mapping it to a situation model and producing triples that are stored in the sensor and social media information fusion layer. This requires annotating the data based on the description provided in the situation model which includes the descriptions of the sensors used, their location, and their units of measurement that are described by the situation model in [35]. Consider a single record from the raw data as shown in Table 2 below.

The corresponding transformed record based on the situation model description from [35] is:

*{‘unit’: ‘http://qudt.org/1.1/vocab/unit#MeterPerSecond’, ‘unit_txt’: ‘degreeAngle’, ‘unit_symbol’: ‘m/s’, ‘cdt_type’: ‘ucum’}*
*wind_gust*
*<http://www.w3.org/ns/sosa/sensor/86338> a sosa:Weather_Station ;*
*sosa:observedProperty <http://www.w3.org/ns/sosa/observableProperty/wind_gust> ;*
*sosa:hasFeatureOfInterest <http://www.w3.org/ns/sosa/FeatureOfInterest/wind> ;*
*sosa:madeBySensor <http://www.w3.org/ns/sosa/Sensor/windspeed_sensor> ;*
*geo:lat “−37.8255”;*
*geo:long “144.9816”;*
*senso:hasCity “melbourne” ;*
*senso:hasPlace “melbourne (olympic park)” ;*
*sosa:resultTime “2021-11-29 19:20:00”^^xsd:dateTime ;*
*sosa:hasSimpleResult “9 “^^cdt:.*

The social media dataset used in [35] was used for evaluating the semantic integration in the CPSA platform. Consider an example social media posting. “Another couple of toasty hours on the bike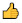 @ Nimmons Bridge https://t.co/K23JxdCBBU, accessed on 6 December 2022”. This posting was posted from Newtown in Australia. We implemented the transformation process described in [35] and the corresponding transformed record for the social media posting is shown below

Tweet country: Australia
Tweet place name: Newtown
Closest weather station: BALLARAT
Location mentioned: [48]
Observable property: temperature
Similarity score, 53.0
Person count: 1
*<http://www.w3.org/ns/sosa/Sensor/1496441118602452997*, accessed on 6 December 2022*> a sosa:Sensor;*
*rdfs:label “Social_Sensor_from_BALLARAT”;*
*sosa:observes <http://www.w3.org/ns/sosa/observableProperty/temperature*, accessed on 6 December 2022*>;*
*sosa:madeObservation <http://www.w3.org/ns/sosa/Observation/temperature_observation_from_BALLARAT*, accessed on 6 December 2022*>;*
*sosa:resultTime “2022_02_23_11_05_39”^^xsd:dateTime;*
*senso:isReportingOn “temperature”;*
*senso:similarityStrength “53.0percent”;*
*senso:hasPersonCount “1”;*
*senso:hasmentionedLocation “*[48]*”;*
*senso:hasCity “BALLARAT”.*

The query response times were used to test the performance of querying engines. For instance, 4.535 s were used to count 71,208 triples. From 71,208 triples the various types of IoT sensors that are reporting were identified in 0.038 secs. Including extracting their names and the name of their weather for a specific day. Queries based on spatial search, such as geocoordinates also performed well and used 0.035 s, whereas for retrieving observations of IoT sensors reporting wind gust information the query took 0.175 s.

#### 8.5.2. Reliable Handling of Large Volumes of IoT Sensor and Social Media Data

The proposed CPSA platform reliably handles huge volumes of data with high velocity and variety from IoT sensor and social media spaces and also to ensure data availability at all times. The likelihood of failure in traditional situation awareness systems increases with an increase in the demand for data requirements especially when the data is being accessed via the cloud. The data needs to be distributed via an efficient cloud architecture comprising of EC2 instances that can help in managing the data. However, this also leads to an increase in the possibility of failure when the number of EC2 instances increases and in the event of a disastrous situation where there are multiple IoT and social media data sources, the likelihood of failure is more for reasons such as physical damage, exhausted batteries, or failure of communication channels, etc. [1].

In this paper, we have utilised the distributed nature of an Apache Kafka cluster and realised it on the open stack cloud for reliably handling of large data volumes when harvesting data from multiple IoT sensor and social media data sources. Apache Kafka works by spreading the data in multiple partitions across different EC2 instances within the cluster. This allows multiple copies of data to be made and maintain copies of the data across different EC2 instances on the cloud. For example, in the data-harvesting engine of the proposed CPSA platform described in Section 5, we made three copies of partitions, i.e., using a replication factor of 3 and stored them on different EC2 instances within the cluster. The replication factor identifies the number of copies of the data. The leader of the topic where the data is stored maintains the first copy and the followers copy the data from the leader. All the copies are configured to be in sync with the leader and the synchronisation is achieved by configuring the property of in-sync replicas in Apache Kafka. This enabled us to tolerate up to two failures, i.e., if two EC2 instances out of three in the cluster go down the system is still able to harvest the data and ensure data availability.

The data-harvesting layer uses the Kafka Producer applications to ingest data from IoT sensors and social media information spaces. We designed three producer applications to read data from various data from IoT and social media data sources. A producer application to harvest weather-related observations into the weather_record_producer topic, an incident producer which produces incidents reported by authoritative government data sources such as country fire authority Victoria and a tweet-producer which produces tweets from Twitter. To ensure load balancing, the producer applications interact with Apache Kafka and produce data to the Kafka brokers in a round robin manner. The load is balanced across multiple Kafka brokers based on the number of partitions. In this paper, we have used the terms producer applications and producers interchangeably.

The social media data for this study was harvested by leveraging the twitter-v2 academic research API from the Twitter platform. In this paper, we aim to evaluate the CPSA platform using real-time data. For these reasons, we configured the topics settings *retention.ms* to hold the data for 7 days in the logs before the log segments are deleted to free up space. Then, we harvest the real-time weather observations of weather sensors from bureau of meteorology (BOM) into multiple topics at every interval such as 10 s, 1 min, 10 min, etc. Using the specific point coordinates (latitude and longitude) for each of the weather stations in Victoria, we then created a set of rules for the producer applications to search for tweets within twenty-five miles of each of these geocoordinates. Then, we filtered tweets from Twitter at every 1 min and stored them in different topics to evaluate the fault tolerance of the data-harvesting layer in CPSA platform. We also trained a weather-prediction model using the real-time data from BOM sensor data and deployed the model in streaming data from BOM. The details of the weather feeling predictions model are out of the scope of this paper. The result of the prediction is stored in the Kafka topic: *bom_weather_predictions_topic*. The results are exported as JSON records which can be plugged into any visualisation or analysis engine. Each partition within the topics mentioned above maintains Kafka offsets which are integer IDs assigned to partitions indicating the position within a partition for the next message that is to be sent Producers.

**Figure 4:**
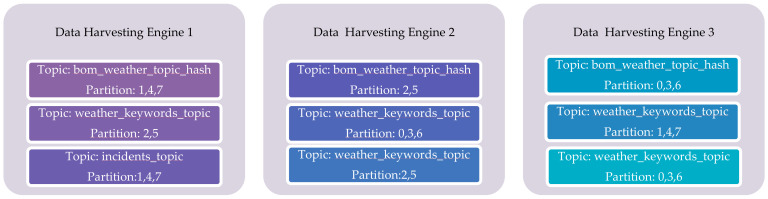
Providing Fault Tolerance using brokers, topics, replicas.

Figure 4 shows the distribution of some of the topics, their replicas across the three brokers in the data-harvesting cluster. We have used both topic level metrics such as messages in per topics, total produce request rate per topic, bytes in per topic, and broker-level metrics such as bytes in/out per broker, total fetch request per broker, etc., to monitor the performance of the data-harvesting layer. We designed and implemented a health-monitoring engine based on Prometheus and Grafana on a standalone EC2 instance in the CPSA platform. This engine consistently collects metrics from Kafka endpoints in the data-harvesting layer. Kafka uses Java management extensions (JMX) and exposes JMX metrics over a HTTP endpoint which are then consumed by Prometheus. Prometheus uses the openmetrics format which is an initiative of the CNCF sandbox project standards. Grafana is an open-source dashboarding tool that uses the metrics put in the pipeline by Prometheus to visualise [67] the cluster health.

**Figure 5:**
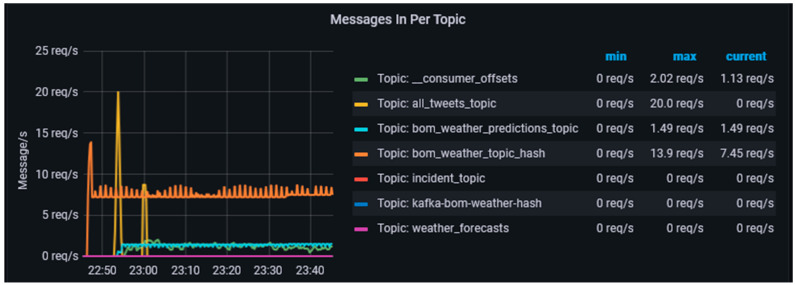
Messages in Per Topic identifying the data volume coming into the data-harvesting cluster.

**Figure 6:**
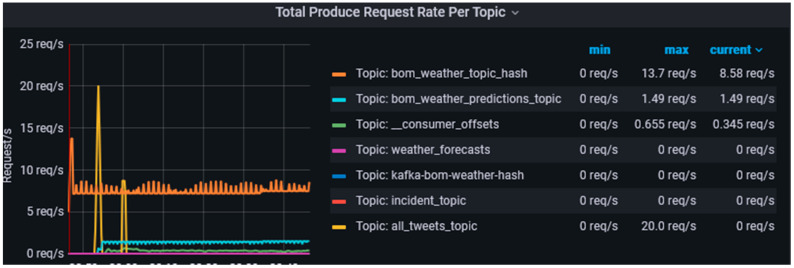
Producer Request Rate indicating ability of the platform to handle spikes.

The *messages in* metric is a topic-level metric which provides information on the mean rate and one-minute rate of incoming messages per second to each topic. As seen in Figure 5, this metric determines the data volume coming into the data-harvesting cluster. The size of this metric as the volume and frequency of messages increases. We can also notice that at about 23.55 h, there was a huge spike in the volume of messages coming in. Further, as seen in Figure 6, the CPSA platform also remains stable under spiky conditions, when harvesting data from various sources which are connected to various topics. However, as the volume and frequency of messages flowing into the topic increase, the size of this metric grows to reflect these. This metric also helps in determining the load on each of the topics which help in estimating the processing limits of the data-harvesting cluster. This information can then be used to determine if the cluster capacity needs to be scaled to handle the increasing demand. This metric can also be used to determine the amount of load is being generated by individual topics which can be achieved either by introducing new nodes into the data-harvesting cluster or by upgrading the capacity of existing nodes in the data-harvesting cluster.

The *request-rate* metric provides information on the average number of requests that are sent per second from the producer applications to the broker which serves as an indicator of the volume of messages generated by the producer applications when harvesting the data. We created various producer applications which harvest data from authoritative data sources, the bureau of meteorology, Twitter, etc. Figure 6 shows that the data-harvesting layer can efficiently handle multiple producer applications in various conditions, spikes in data, constant velocity data, and no data.

**Figure 7:**
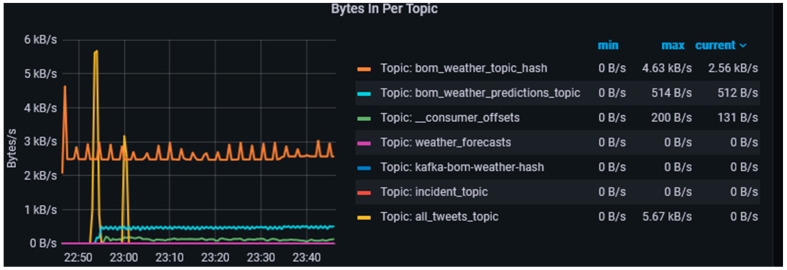
Bytes into each topic to determine the load being generated by each topic.

The *Bytes In* metric shown in Figure 7 provides information on the mean rate and per minute rate at which bytes arrive per second into individual topics. However, as the volume and frequency of messages flowing into the topic increase, the size of this metric also grows like the *messages in* metric to reflect these. The benefit of using this metric is it helps in determining the cluster capacity and in the event of a full capacity whether end-to-end compression of messages is required. amount of load is being generated by individual topics. We configured the producers to follow a snappy compression mechanism. The default batch size for messages in Kafka is 16 kb [68]. We increased the batch size to 64 kb to ensure the producers create batches of messages when multiple batches are being written into the same partitions and the request rate remains consistent. This helps in achieving a stable performance and throughput of the brokers [68] in the data-harvesting layer.

**Figure 8:**
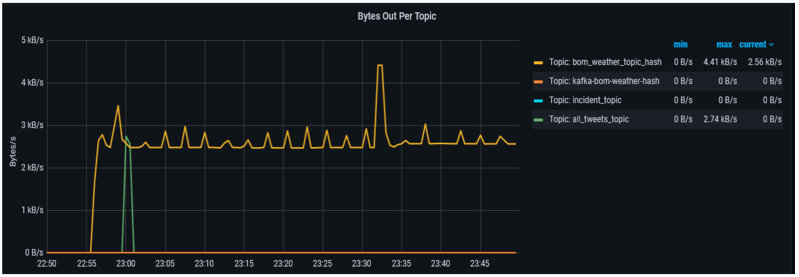
Bytes out from each topic to determine the load being generated by each topic.

The *Bytes Out* metric shown in Figure 8 provides information on the mean rate and per minute rate at which bytes depart per second from individual topics. However, as the volume and frequency of messages flowing out of the topic increase, the size of this metric also grows like the *bytes in* the metric to reflect these. In Figure 8 we can see that multiple consumer applications consuming the messages are able to read the messages without affecting the broker workload.

**Figure 9:**
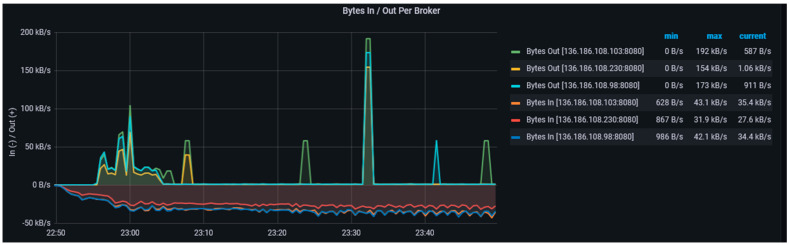
Bytes In/Out from broker indicating the overall throughput.

The *BytesInPerSec/BytesOutPerSec* is a broker-level metric and describes the amount of data each broker written from producers and the amount of data consumed by the consumer applications from the broker which provides information on the broker throughput and helps in diagnosing network bottleneck issues. As seen in Figure 9 the overall throughput on the data-harvesting cluster is stable based on the configurations discussed in Section 8.1 and Section 8.2.

**Figure 10:**
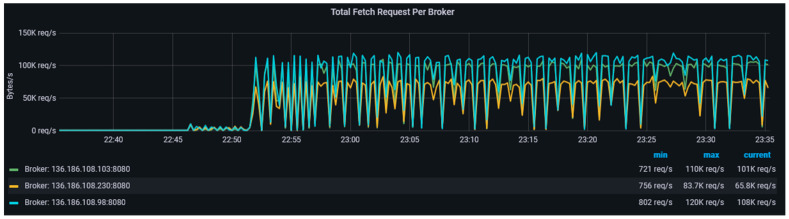
Frequency of requests to produce, consume per broker.

The total fetch requests per broker identify the frequency of requests from producers, consumers, and followers in the data-harvesting cluster and is shown in Figure 10. This information in real time is captured at every minute. The rate of requests should be monitored to ensure effective communication between the producers and consumers in the data-harvesting cluster. We ensured that this request rate remains stable by setting a batch size of 64 kb on the producer applications. Further, in a healthy Kafka cluster, the number of *insyncreplicas* (ISRs) are exactly equal to the total number of replicas. If partition replicas fall too far behind their leaders, Kafka removes the follower partition from the *insyncreplicas* pool, and the value of *IsrShrinksPerSec* increases rapidly. Under-replicated partitions metrics are a strong indicator when one or more brokers become unavailable for various reasons which include taking off the broker for maintenance.

When we restarted the brokers to simulate brokers going off from the cluster, as seen in Table 3, the value of *UnderReplicatedPartitions* increased from 0 to 295. However, as seen in Table 4, when the brokers were back after being restarted, all the 295 under replicated partitions were synced across the brokers indicating the reliable handling of large volumes of data. The design of our three-node data-harvesting cluster allows reliable data handling even when two brokers are taken off and still continue to harvest data without degrading the performance. We now turn off two brokers one of which, 136.186.108.103 is the controller of the data-harvesting cluster and 136.186.108.230, a follower in the data-harvesting cluster and to test the tolerance of the platform to faults. A controller in Apache Kafka is responsible for smooth and resilient functioning of the Kafka cluster. In a cluster of N brokers, there is always only one controller. When the data-harvesting cluster boots up, the first node to boot is assigned the role of the controller by Apache zookeeper and an ephemeral node/controller is created within zookeeper and the rest of the brokers keep a watch on this ephemeral node. The controller is responsible for maintaining a list of partition leaders and also in coordinating the transitions in leadership whenever a partition leader becomes unavailable. When the controller is down, the rest of the brokers will be notified by a zookeeper and the zookeeper begins the election of a new controller. As seen in Table 3 from the *ActiveControllerCount* metric, although two brokers were taken off the active controller count still remained at 1. However, when the initial controller (136.186.108.103) was down, the zookeeper choose a new controller in 136.186.108.98 as it was the only available broker in the cluster. When a partition remains without an active leader, that partition will be inaccessible, and the consumers and producers reading and writing data from that partition will be blocked until the leader becomes available.

The sum of the *ActiveControllerCount* metric always needs to be equal to 1, indicating a healthy cluster. When we took off two brokers for evaluation, the metric *OfflinePartitionsCount* went from 0 to 7 when the two brokers were taken off. This metric reports the number of partitions without an active leader. As the partition leaders are responsible for performing read and write operations, any non-zero value indicates that there are service interruptions. As soon as the brokers were restarted the *UnderReplicatedPartitions* have come down to 0 and the leaders and followers were reassigned by the zookeeper as soon as the brokers were back, the *OfflinePartitionsCount* returned also returned back to 0 indicating the capability of the data-harvesting cluster to recover from faults. In Table 4, we can see that the metrics have returned to their normal values and the data CPSA platform was able to recover from faults in the data-harvesting cluster without any disruptions.

## 9. Discussion of the CPSA Platform

The development of the CPSA platform for improving situation awareness capabilities using situation models that help in integrating sparse IoT sensor data and social media postings has the potential to provide richer and more accurate situation awareness information. We provided extensive experimental results derived from the evaluation of the CPSA platform when providing situation awareness using both IoT sensors and social media data. Real-time, streaming weather sensor data from the bureau of meteorology and social media data is harvested for demonstrating the validity and evaluation of the proposed CPSA platform concept. Overall, the results are promising and seem to encourage the adoption of the CPSA platform in such environments. In Section 5, Section 6 and Section 7, we demonstrated the ability of the CPSA platform to semantically describe and integrate the information extracted from the sensor and social media spaces and its ability to intersect information from these spaces using situation models to enrich situation awareness. In Section 8, we have also demonstrated that the CPSA platform can reliably handle large data volumes when harvesting data from various IoT sensors and social media data sources and also when deploying pre-trained machine learning models to make weather predictions in near real time. In doing so, it is also important to manage the health of the machines that are involved in the data processing. To manage the health of the data-harvesting cluster, the zookeeper design allows the cloud instances in the cluster to be connected to different zookeeper servers with the leader responsible for writing and followers for read operations, respectively.

The data-harvesting layer allows connection to various sensor and social media data sources such as automatic weather stations, Twitter, and other authoritative data sources such as the CFA, etc. When extracting data from Twitter, dynamic queries were used for the identification and extraction of relevant data. This included matching the data from social media data spaces using the geolocation parameters from the sensor data for retrieving tweets that are geotagged to these location coordinates. We leveraged kafka offsets for storing the incoming data streams (messages) and the kafka offsets that store this incoming data from sensor and social media data sources can never be updated or swapped. New data cannot be written on the same offset, providing guarantees for data ordering which are critical in situation awareness applications. However, message ordering in systems using kafka is guaranteed only within a specific partition and not across partitions.

The feature extraction and semantic mapping layer are optimised to provide efficient support for distributed processing when handling high data volumes and data that requires incremental computation and streaming data. This layer within the CPSA platform supports dynamic workloads for distributed applications written in languages such as Scala, Python R, and Java. The configuration of machines operating in this layer of the CPSA platform, discussed in Section 8.3, allows them to run either as individual machines or using various cluster deployment modes such as standalone, YARN, and Kubernetes. Further, when handling huge volumes and a variety of data harvested from sensors and social media postings, the sensor, and social media fusion layer within the CPSA platform supports the use of open-source semantic frameworks when using ontology-based situation models for semantically describing situations and integrating the data from the sensor and social media information spaces. As we discussed in Section 8.5.2, and from the results in Table 3 and Table 4 the design of the data-harvesting cluster allows the CPSA platform to reliably handle data even when only one machine is available in the cluster when the rest are taken off for maintenance, upgrades, etc., and still continue to harvest data without degrading the performance.

## 10. Conclusions and Future Work

The CPSA platform described in this paper can be used to enrich situation awareness using both IoT sensors and social media data. In this paper, we demonstrated how the CPSA platform can be used to reliably handle large volumes of IoT sensor and social media data streams and also perform their semantic integration. However, during disaster situations, it is possible that the CPSA platform needs to be scaled to handle spikes. The proposed CPSA platform can be manually scaled in the event of spikes and when there is no disaster situation, i.e., in normal situations, the cloud resources of the CPSA platform are underutilised. In future research, we aim to provide automatic provisioning and scaling of cloud resources to resolve the underutilisation of resources when enriching situation awareness using both IoT sensors and social media information spaces. The capabilities of the CPSA platform to support semantic integration on the sensor and social media data have allowed us to deliver the ability to infer more knowledge using situation models. Furthermore, this knowledge can, at times, be complimentary and contradictory, and it is also necessary that further performance evaluations are performed in these cases to ensure the best utilisation of system resources. There is also a need to explore open-source ecosystems, such as Apache zeppelin, that can be integrated within situation-awareness systems to build real-time dashboards to visualise real-time situation awareness.

## Figures and Tables

**Table 1:** Situation Awareness Applications, their data sources, and use of semantics.

Author	Situation-Awareness Application	Sensor Data	Social Media Data	Both Sensor and Social Media Data	Semantics
**[1]**	Smart City	✓	✓	✓	X
**[6]**	Floods	✓	✓	✓	X
**[7]**	Smog	✓	✓	✓	X
**[16]**	Floods	✓	✓	✓	X
**[18]**	Smart Transportation	✓	X	X	✓
**[20]**	Cyber Crime	X	✓	X	✓
**[22]**	**Natural Hazards**	✓	✓	✓	X
**[23]**	Floods	✓	✓	✓	X
**[29]**	Smart Transportation	✓	X	X	✓
**[30]**	Smart spaces	✓	X	X	✓
**[31]**	Smart Homes	✓	X	X	✓
**[32]**	Network Security	✓	X	X	✓
**[33]**	Disasters	X	✓	X	X
**[34]**	Smart Parking	✓	X	X	✓
**[35]**	Weather	✓	✓	✓	✓

**Table 2:** Sample observation from the Melbourne Olympic Park weather station [35].

Attribute	Value
place_name	melbourne
station_name	melbourne (olympic park)
temperature	23.2
humidity	56
wind_direction	SSW
wind_speed	6
wind_gust	9
pressure	1014.3
time_reported	29/11/2021 19:20
lat	−37.8255
long	144.9816
station_id	86338

**Table 3:** Broker and Partition status when brokers are taken off from the cluster.

Brokers in Cluster	Brokers Online	Brokers Offline	Active Controller	Active Controller Count	Followers	Under Replicated Partitions	Offline Partitions
136.186.108.98 136.186.108.230 136.186.108.103	136.186.108.98	136.186.108.230 136.186.108.103	136.186.108.98	1	-	295	7

**Table 4:** Broker and Partition status when brokers are back in to the cluster.

Brokers in Cluster	Brokers Online	Brokers Offline	Active Controller	Active Controller Count	Followers	Under Replicated Partitions	Offline Partitions
136.186.108.98 136.186.108.230 136.186.108.103	136.186.108.98 136.186.108.230 136.186.108.103	-	136.186.108.98	1	136.186.108.230 136.186.108.103	0	0

## Data Availability

Not applicable.
